# Supraspinal and Afferent Signaling Facilitate Spinal Sensorimotor Network Excitability After Discomplete Spinal Cord Injury: A Case Report

**DOI:** 10.3389/fnins.2020.00552

**Published:** 2020-06-22

**Authors:** Alena Militskova, Elvira Mukhametova, Elsa Fatykhova, Safar Sharifullin, Carlos A. Cuellar, Jonathan S. Calvert, Peter J. Grahn, Tatiana Baltina, Igor Lavrov

**Affiliations:** ^1^Institute of Fundamental Medicine and Biology, Kazan Federal University, Kazan, Russia; ^2^Children’s Republican Clinical Hospital of the Ministry of Health of the Republic of Tatarstan, Kazan, Russia; ^3^Dicom Clinic, Kazan, Russia; ^4^Centro de Investigación en Ciencias de la Salud, Universidad Anáhuac México, Huixquilucan, Mexico; ^5^Department of Neurologic Surgery, Mayo Clinic, Rochester, MN, United States; ^6^Department of Physical Medicine and Rehabilitation, Mayo Clinic, Rochester, MN, United States; ^7^Department of Biomedical Engineering, Mayo Clinic, Rochester, MN, United States; ^8^Department of Neurology, Mayo Clinic, Rochester, MN, United States

**Keywords:** spinal cord injury, AIS-A, discomplete spinal cord injury, spinal cord stimulation, sub-lesional spinal circuitry

## Abstract

**Objective:**

In this study, we evaluated the role of residual supraspinal and afferent signaling and their convergence on the sublesional spinal network in subject diagnosed with complete paralysis (AIS-A).

**Methods:**

A combination of electrophysiologic techniques with positional changes and subject-driven reinforcement maneuvers was implemented in this study. Electrical stimulation was applied transcutaneously at the T9-L2 vertebra levels and the spinal cord motor evoked potentials (SEMP) were recorded from leg muscles. To test the influence of positional changes, the subject was placed in (i) supine, (ii) upright with partial body weight bearing and (iii) vertically suspended without body weight bearing positions.

**Results:**

Increase in amplitude of SEMP was observed during transition from supine to upright position, supporting the role of sensory input in lumbosacral network excitability. Additionally, amplitudes of SEMP were facilitated during reinforcement maneuvers, indicating a supralesional influence on sub-lesional network. After initial assessment, subject underwent rehabilitation therapy with following electrophysiological testing that reviled facilitation of SEMP.

**Conclusion:**

These results demonstrate that combination of electrophysiological techniques with positional and reinforcement maneuvers can add to the diagnostics of discomplete SCI. These findings also support an idea that integration of supraspinal and afferent information on sub-lesional circuitry plays a critical role in facilitation of spinal sensorimotor network in discomplete SCI.

## Background

According to the World Health Organization, global estimate of up to 500,000 people sustain a SCI each year (Kumar et al., 2018). Disruption of neural connections between the brain and spinal cord after SCI leads to permanent functional impairment. The American Spinal Injury Association (ASIA) Impairment Scale (AIS) is a widely accepted diagnostic tool for assessment of SCI (Kirshblum et al., 2014). However, the AIS classification of “complete” or “incomplete” loss of function is not sensitive with respect to severity of tissue injury, nor does it indicate the presence of sub-functional connectivity across the injury in those diagnosed with complete AIS-A paralysis (Awad et al., 2015). Despite clinical diagnosis of complete absence of voluntary control after SCI, prior evidence suggests a majority of injuries contain sub-functional connections that are capable of transmitting supra-spinal influence on spinal circuitry excitability below the injury (Dimitrijevic et al., 1984, 1987). This injury profile is known as “discomplete SCI” (Dimitrijevic et al., 1987). Specifically, in a cohort of subjects diagnosed as motor complete (AIS-A or B), attempts to volitionally initiate foot movements resulted in 89% of muscles generating EMG activity, suggesting some level of voluntary control over muscle activity (Moss et al., 2011). Other studies reported anatomical and electrophysiological findings, indicating that some ascending and descending fibers remain intact across the damaged area of the spinal cord in AIS-A subjects (Kakulas, 1988). Unfortunately, currently available electrophysiological and imaging tools are insufficient in identifying discomplete SCI (Nicotra and Ellaway, 2006). Animal studies and clinical trials results indicate that spinal cord electrical stimulation alone (Dimitrijevic et al., 1998; Gerasimenko et al., 2001; Lavrov et al., 2006, 2008, 2015; Harkema et al., 2011; Cuellar et al., 2017; Grahn et al., 2017; Shah and Lavrov, 2017) and in combination with medications (Gerasimenko et al., 2015) or/and locomotor training (Gerasimenko et al., 2017), significantly improved sensorimotor and autonomic functions after SCI. These data suggest that advanced diagnostic tools need to be developed to identify functionally silent connections for targeted engagement of sub-lesional spinal circuitry via emerging neuromodulatory therapies (Minassian et al., 2016; Taccola et al., 2018; Islam et al., 2019). Here, we present a case report of the patient with an SCI classified as AIS-A with complete loss of motor and sensory function below the injury, who demonstrated the residual supraspinal and afferent signaling on the sublesional spinal network during combination of electrophysiologic techniques, changes in body position, and subject-driven reinforcement maneuvers (see Supplementary Material).

## Case Presentation

**FIGURE 1:**
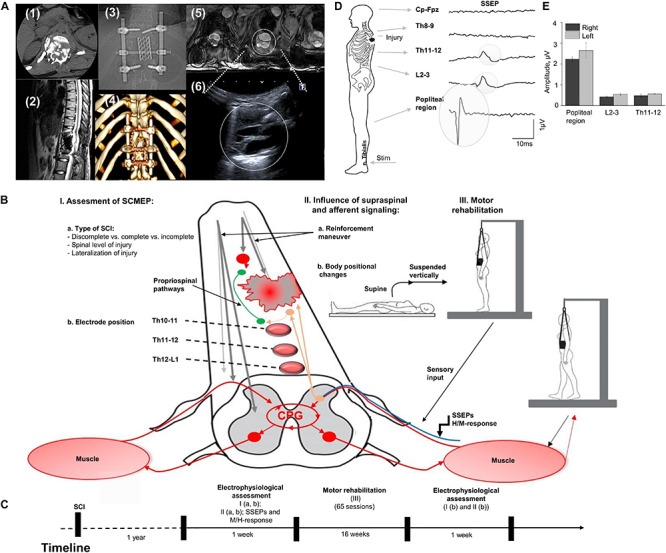
**(A)** Initial evaluation with CT cross-section at injury level (1); the sagittal MRI view of the thoracic spine with the area of SCI; (3) X-ray view of the spine fixation structure; (4) 3D reconstruction of vertebras with areas of laminectomy and spine fixation structure, circles indicate position of the ultrasound sensor in the projection of laminectomy; (5) three transverse MRI sections of the spinal cord at the injury level (T1 weighted lesion); (6) visualization of the spinal cord at injury level with ultrasound technique. **(B)** Study design with approach of SCI evaluation in human. Assessment of SEMP (I) with the role of injury type (a) and electrode position (b); the influence of supraspinal and afferent information (II) tested with reinforcement maneuvers (a) and positional changes (b); and the role of motor rehabilitation (III). **(C)** Timeline of the study. **(D)** An example of somatosensory evoked potentials (SSEP) recorded with stimulation of the n. Tibialis with recording electrodes located at the Cz-Fpz, Th8-9, Th11-12, L2-3, and popliteal region. Average of 800 responses presented for each location. Gray circles indicate the SSEP at the Th11-12, L2-3, and popliteal region. **(E)** SSEP amplitude at popliteal region, L2-3, and Th11-12 level during stimulation on the right and left n. Tibialis (*n* = 3).

**FIGURE 2:**
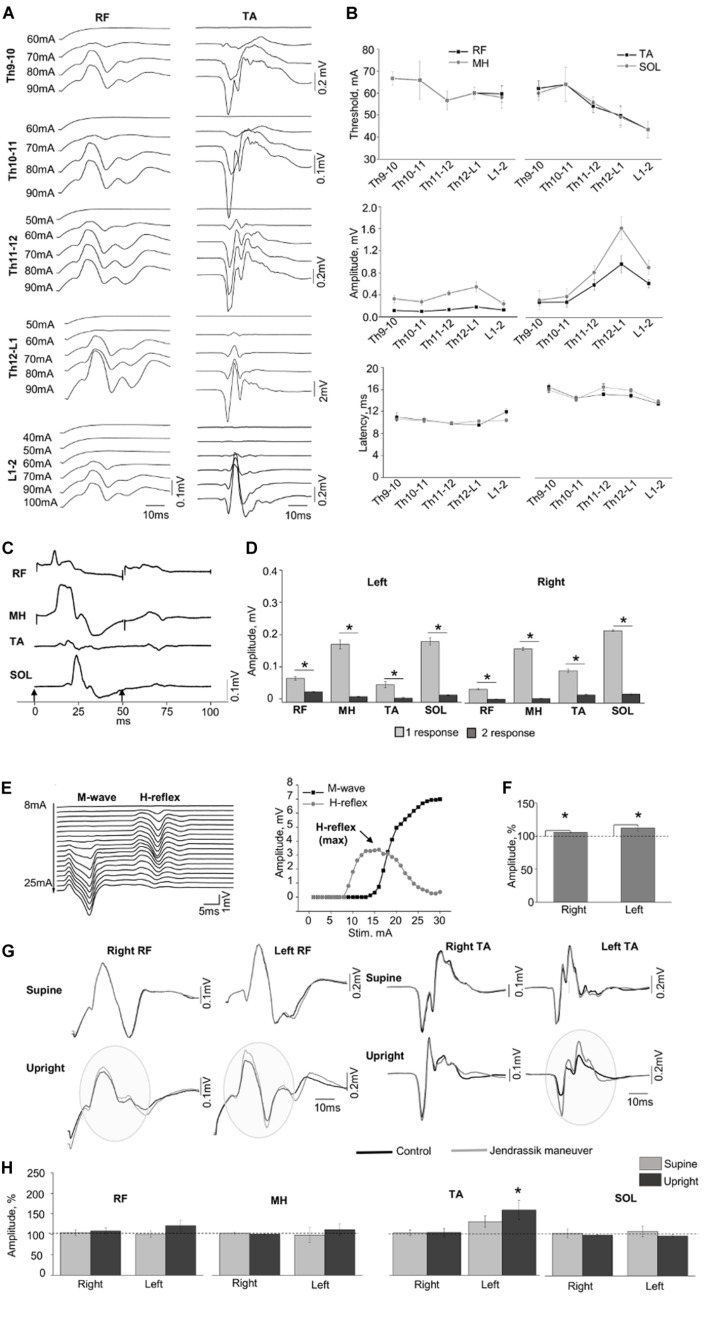
**(A)** Examples of SEMP recorded from proximal (RF) and distal (TA) muscles during stimulation at Th9-10, Th10-11, Th11-12, Th12-L1, and L1-2 levels, in supine position. **(B)** Changes in the thresholds, maximal amplitudes, and the latency of the SEMP recorded from proximal (RF and MH) and distal (TA, SOL) muscles with stimulation applied at Th9-10, Th10-11, Th11-12, Th12-L1, and L1-2 levels. **(C)** Examples of the SEMP recorded from RF, MH, TA, and SOL with paired pulses stimulation (interstim interval of 50 ms) at Th11-12 level. Black arrow indicate the moment of the stim. **(D)** The amplitudes of the SEMP recorded from right and left side during paired stimulation at Th11-12 level. **(E)** Examples of M wave and H-reflex recorded form SOL muscle at stimulation intensity varied from 8 to 25 mA with increment of 1 mA. Recruitment curves of the M wave (black line) and the H-reflex (light gray line) presented on the right. **(F)** The amplitudes (%) of the H-reflex recorded from right and left side (*n* = 10) during performance of Jendrassik maneuver (gray bars). Dotted lines indicate the control values of the H-reflex (100%). **(G)** Examples of the SEMP recorded from RF and TA during stimulation at Th12-L1 without (black line) and with Jendrassik maneuver (gray line) in supine and in upright (less than 30% body weight support) positions. Gray circles indicate the facilitation of the SEMP bilaterally RF, and in left TA by Jendrassik maneuver. **(H)** The amplitudes (%) of the SEMP recorded from right and left proximal (RF and MH) and distal muscles (TA and SOL) with stimulation at Th12-L1 during performance of Jendrassik maneuver in supine (light gray) and upright (less than 30% body weight support) positions (dark gray) in subject with SCI (*n* = 4). Dotted lines indicate the control values of the SEMP (100%). Difference marked with an asterisk indicates significance (*p < 0.05).

**FIGURE 3:**
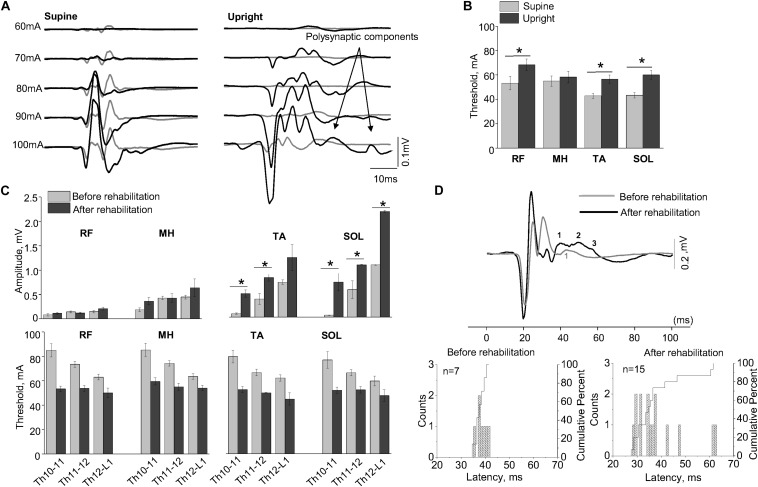
**(A)** Examples of the SEMP recorded from TA muscle during stimulation at Th11-12 level in supine and upright (less than 30% body weight support) positions after the first verticalization before (gray lines) and after rehabilitation therapy (black lines). **(B)** The thresholds of the SEMP recorded in supine (gray lines) and upright (less than 30% body weight support) position after the first verticalization (black lines) in TA, SOL, RF, and MH (*n* = 6). **(C)** Changes in amplitude and threshold of SEMP recorded from RF, MH, TA, and SOL in supine position before (gray) and after (black) rehabilitation therapy (*n* = 4). **(D)** Example of the SEMP recorded from TA during stimulation at Th11-12 level in upright position (less than 30% body weight support) before (gray line) and after rehabilitation therapy (black line). The black and gray numbers indicate the number of polysynaptic components of the SEMP. Histograms and cumulative percentage of latencies (ms) of polysynaptic components of the SEMP recorded from TA before and after rehabilitation. Counts – frequency of occurrence of latencies of LR in interval of 1 ms. Cumulative percent – cumulative percentage of frequency of occurrence of latencies of LR in interval of 1 ms. Difference marked with an asterisk indicates significance (*p < 0.05).

The participant is a 21-year-old woman (163 cm, 55 kg) with no previous disease with Th12 vertebra fracture associated with spinal cord compression and spinal cord injury at the level Th11 (ASI A), multiple rib fractures, contused lung, traumatic hiatal hernia, kidney contusion, followed by paraplegia, sensory loss, loss of bladder and bowel control. Urgently, she underwent hepatorrhaphy, and 5 days after injury, the decompression spine surgery at the level Th12, followed by reduction spondylodesis Th11-L1 (Figures 1A-1,2,3). A computed tomography scan (CT) was performed before surgery and magnetic resonance imaging (MRI) was captured post-surgery, although, some distortion was apparent due to spinal fixation hardware (Figures 1A-2,5). Additionally, injury site was assessed with ultrasound (Figure 1A-6). One year after SCI, participant was enrolled into the study and underwent a re-evaluation of neurological functions below the lesion along with electrophysiologic assessment with positional changes and subject-driven reinforcement maneuvers. The neurological assessment was consistent with paraplegia with decreased muscle tone in proximal leg muscles and increased in distal muscles, neurological level of injury Th11. Light touch sensory loss from the level Th12 bilaterally, pinprick sensory loss from the level Th12 from the left side and L1 from the right side, joint position sense loss from the level Th12, loss of bladder control (uses clean intermittent catheterization, residual urine volume: 200–400 ml), loss of bowel control. Figure 1B summarizes tested in this report electrophysiological assessment: (I) examination of spinally evoked motor potentials (SEMP) to transcutaneous stimulation (tSCS) applied at Th9-10, Th10-11, Th11-12, Th12-L1, L1-2 levels; (II) the evaluation of the supraspinal influence and afferent signaling by assessment the effect of reinforcement maneuver (Jendrassik maneuver, JM) and positional changes. First, the effect of the JM was evaluated during testing H-reflex in supine position. Then, we investigated the combination of JM and afferent signaling with tSCS in supine and upright (less than 30% body weight support) positions (Apte et al., 2018). The visual assessment of the leg muscle activation during JM was evaluated in supine and in vertically suspended (100% body weight support) positions; (III) the impact of the motor rehabilitation on facilitation of the mono- and polysynaptic spinal cord circuitry. During the initial electrophysiological assessment, the subject with SCI was evaluated with techniques I and II (Figures 1B,C). After the initial assessment 65 rehabilitation sessions, approximately 45 min each, consisting of trainer-assisted standing and weight supported stepping were performed over 16 weeks, with the following electrophysiological assessment (Figure 1C; see Supplementary Material).

## Results

### Electrophysiological Assessment of the Discomplete SCI

#### Evaluation of the Continuity of the Posterior Columns

The amplitude of the SSEP at popliteal region, L2-3, and at Th11-12 levels on the low extremities at each recording location is presented on Figure 1 E and C. SSEPs were not detected at Th8-9 and Cz-Fpz levels located above the SCI (Figure 1D, two uppermost traces).

#### Evaluation of Spinally Evoked Motor Potentials at Different Spinal Levels

Figure 2 demonstrates examples of the SEMP in m. rectus femoris (RF) and m. tibialis anterior (TA) obtained at different stimulation intensities (Figure 2A) with threshold, amplitude values, and latency of the SEMP (Figure 2B) during tSCS at Th9-10, Th10-11, Th11-12, Th12-L1, and L1-2 levels (obtained at 100 mA). The order of activation of different muscles was dependent on the rostrocaudal location of stimulating electrodes. The stimulation intensity required to reach the motor threshold was gradually decreased from Th9-10 and Th10-11 to L1-2 in proximal and in distal muscles (*n* = 6, *p* < 0.05). In both distal and proximal muscles, the maximal amplitude of SEMP was gradually increased from T9-10 level reaching the highest value at T12-L1 and then decreased at the L1-2 level (*n* = 6, *p* < 0.05) (Figure 2B). The SEMP average latencies for the distal muscles were 14.90 ± 0.50 ms for the TA, 15.28 ± 0.52 ms for the SOL, 10.53 ± 0.43 ms for the RF and 10.27 ± 0.11 ms for the MH (*n* = 5). The SEMP latency was compatible with the distance between the stimulation level and the muscle and was larger in TA and m. soleus (SOL) and shorter in proximal muscles

RF and medial hamstring (MH) (Figure 2B). The maximal amplitudes of SEMP for proximal muscles were significantly lower compared to distal muscles (*n* = 4, *p* < 0.05). Examples of SEMPs recorded with paired tSCS at Th11-12 level are presented on Figure 2C. It is evident that the SEMPs were depressed with paired spinal cord stimulation (see more method details in Supplementary Material), supporting the reflex nature of the observed responses (*n* = 6, *p* < 0.05) (Figure 2D).

#### Evaluation of the Supraspinal-Spinal Connectivity

The M-wave and the H-reflex were recorded in SOL muscle (Figure 2E). During the JM the amplitude of H-reflex increased to 106.02 ± 0.94% and 111.43 ± 1.84% from control 100% values for the right and left leg, respectively (*n* = 10, *p* < 0.05) (Figure 2F). Figure 2G demonstrates examples of changes in the amplitude of SEMP recorded from RF and TA muscles without and with the JM, tested in supine and upright positions during the spinal cord stimulation at Th12-L1 level. Amplitude of the SEMP during JM was significantly facilitated in the left TA to 153.17 ± 22.45% from control 100% values only in upright position (*n* = 4, *p* < 0.05) (Figures 2G,H, Upright). In other muscles, JM did not change SEMP for either right or left leg (Figure 2H). Amplitudes of SEMP during JM with respect to the control condition were to 103.91 ± 7.14% (supine), and to 108.14 ± 7.79% (upright); in left RF to 101.09 ± 7.90% and to 121.10 ± 13.35%; in right MH to 102.83 ± 2.73% and to 100.87 ± 1.95%; in left MH to 98.53 ± 18.39% and to 111.43 ± 14.76%; in right TA to 102.65 ± 3.04% and 104.51 ± 8.64; in left TA to 129.16 ± 14.15% (supine); in right SOL to 104.28 ± 11.09% and to 99.67 ± 1.55%; in left SOL to 108.45 ± 12.70% and to 97.46 ± 3.81%; from control 100% values. In addition, delayed motor response with great toe extension was repeatedly observed on the right and left leg during JM only in vertically suspended (Supplementary Video S1).

#### The Effect of Rehabilitation Therapy on SEMP

Figure 3A shows examples of SEMP in TA muscle, evoked by stimulation of Th11-12 in supine position (supine) and immediately following the first verticalization (upright) before (gray lines) and after 16 weeks of rehabilitation therapy (black lines). Changes in SEMP (amplitude and threshold) before and after rehabilitation therapy in the supine position presented on Figure 3C. Maximum values of monosynaptic SEMP component in distal muscles before rehabilitation therapy were significantly lower compared to the amplitudes of SEMP after rehabilitation therapy (*n* = 4, *p* < 0.05) (Figure 3C). After subject with SCI underwent rehabilitation therapy, the thresholds of SEMP significantly decreased in all muscles (*n* = 4, *p* < 0.05) (Figure 3C). Polysynaptic components of the SEMP were found in TA muscle after rehabilitation therapy particularly in upright position. The cumulative analyses of latencies of LR demonstrated that the latencies of the polysynaptic components had wider distribution after rehabilitation therapy in upright position and mostly in TA (Figure 3A, Upright, and Figure 3D).

## Discussion

In this study we evaluated the influence of supraspinal and afferent information on sub-lesional spinal circuitry excitability in subject with AIS-A SCI. The results demonstrate: (1) body position can change the excitability of spinal circuitry and, in combination with reinforcement (Jendrassik) maneuvers, facilitate sub-functional connectivity, indicating the discompleteness of injury; (2) the effect of motor rehabilitation therapy on spinal circuitry excitability with respect to SEMP (Figure 1A).

### Assessment of SEMP After SCI in Lumbosacral Level

Considering the importance of the functional state of sublesional circuitry in evaluation of spared subfunctional fibers, we hypothesized that the characteristics of SEMP with tSCS can indicate excitability across several spinal cord segments (Minassian et al., 2007; Dy et al., 2010) and, accordingly, provide detailed information on motoneuronal pools related to multiple muscles (Courtine et al., 2007). The results of this case report, combined with previous reports (Troni et al., 2011; Roy et al., 2012; Krenn et al., 2013; Sayenko et al., 2015) suggest that, the tSCS at different spinal levels can modulate the activation order of proximal and distal muscles. The different latency of proximal (RF and MH) and distal muscles (TA and SOL) can be explained by the difference in anatomical distribution of the motor pools and the distance between the place of stimulation and muscle. The amplitude of the SEMP with caudal shifting of the stimulation electrodes was gradually increased in distal muscles, meanwhile proximal muscled showed minimal changes in amplitude compared to the amplitude of response in distal muscles that can be explained by subject’s injury level (Th11). Also, the activation order of proximal and distal muscles could be related to different localization of motoneuronal pools (Phillips and Park, 1991) or current flow passage across several layers of back and spine tissues and anatomical curvatures (Hofstoetter et al., 2014).

### The Role of the Supraspinal and Afferent Information in Assessment of SCI

#### The Influence of Reinforcement Maneuvers on H-Reflex and SEMP

Previous studies indicated that supraspinal influence may have different effect on motoneurons and interneurons (Sabatino et al., 1995) and, therefore, modulation of mono- and polysynaptic responses can be a sensitive assessment tool of the spinal cord circuitry functional state after SCI. Given the nature of the H-reflex and the reflex components of the SEMP, it can be expected that the JM contribute in activation of downstream effects on spinal neuronal circuit. JM (Jendrassik, 1883), was used as a reinforcement to study spinal cord evoked responses in control subjects and in subjects with SCI (Dimitrijevic et al., 1977). One of the possible mechanisms of the effect of JM on spinal cord excitability was related to reduction of segmental presynaptic inhibition (Zehr and Stein, 1999). Our results indicate that JM can alter the H-reflex in supine position and SEMP in upright positioning the subject with discomplete SCI. At the same time, SEMP were affected primary in the left TA. As opposed to the SOL H-reflex, which is a monosynaptic response in a single muscle, the SEMP evoked by tSCS is related to a complex spinal network. Thus, facilitation of SEMP during JM may reflect the results of complex intraspinal and intersegmental interaction compared to monosynaptic response related to a single motor pool during H-reflex.

#### The Influence of the Positional Changes on the SEMP

The SEMP were previously studied applying tSCS in healthy subjects and in subjects with SCI, tested in various positions: supine (Minassian et al., 2007; Hofstoetter et al., 2018), upright, and during gait modulation (Minassian et al., 2015). It was demonstrated that in individuals with AIS-A and-B positional changes (supine vs. standing) can provide different modulation of SEMPs components (Sayenko et al., 2014). In this study, transition from supine to upright position facilitated the amplitude of SEMP components that could be related to changes in sensory information from mechanoreceptors affecting the spinal circuitry excitability (Harkema et al., 1997). Presynaptic inhibition of Ia afferents on the motoneuron is considered to be controlled by descending tracts and the level of presynaptic inhibition input in SCI subjects declines compared to control subjects, contributing to enhancement of spinal reflexes (Calancie et al., 1993). The body position could influence the activation of the afferent and efferent fibers by tSCS (Danner et al., 2016). In contrast to our results, Danner et al. (2016) showed that the thresholds of evoked responses in subjects with intact spinal cord were lowest in upright position and highest in the prone position (Danner et al., 2016). Variations in mono- and polysynaptic responses during tSCS can be related to motoneuronal excitability and also to complex convergence of sensory afferents on spinal reflex pathways (Schomburg, 1990; Sayenko et al., 2014). It was suggested that the afferent information can be integrated by spinal circuitry and result in elevation interneuronal excitability during standing (Harkema et al., 2011; Rejc et al., 2015). Also, Sayenko et al. (2019) demonstrated that tSCS can modulate the lumbosacral spinal networks to facilitate postural control after SCI (Sayenko et al., 2019). Therefore, the characteristics of SEMP cannot be attributed only to a certain motor pools related to the spinal cord circuitry, but rather to specific interplay of multiple peripheral sensory resources and related interneurons (Sayenko et al., 2014). These results indicate that positional changes can facilitate lumbosacral networks and increase the sensitivity of electrophysiological testing for the residual sub- functional connections after SCI.

#### Assessment of SEMP After Motor Rehabilitation Therapy

Complete paralysis of the lower extremities and ability to stand and perform coordinated motor activity could be improved with epidural electrical stimulation (Harkema et al., 2011; Grahn et al., 2017; Gill et al., 2018; Wagner et al., 2018). Our results demonstrate that rehabilitation therapy can facilitate SEMP components, observed after 16-week rehabilitation program in supine and upright positions. It is noteworthy that facilitation of the late response in calf muscles was found in upright position. Similar to animal studies, initiation of rhythmic activity after SCI in human was associated with appearance of late responses (Minassian et al., 2004). As it has been shown earlier, the task-specific training with epidural SCS may reactivate previously silent neural circuits or promote plasticity (Harkema et al., 2011). In addition, tSCS, as well as epidural SCS, can modulate spinal circuitry in humans after SCI that enables sensory inputs to serve as a primary source of neural control of posture and balance (Sayenko et al., 2019). Decreased threshold and increased reflex excitation may be indicative for an increased spasticity. The presence of spasticity below the level of injury in patients with SCI could indicate that related motor pools are relatively preserved (Harris et al., 2007; Gorgey and Dudley, 2008), at the same time, characteristics of SEMP cannot be attributed only to the level of the spinal circuitry excitation and could be a consequence of interplay of multiple peripheral afferent signals and related interneurons (Sayenko et al., 2014). Findings of multiple studies suggest that spinal reflexes increase in patients with SCI and cannot be evaluated unambiguously. Particularly in this study, an increase of the motoneurons’ excitability was not clearly related to spasticity.

## Limitations

The key limitation of this research is that data were collected from one research participant. Another factor that should be considered in this study, is the titanium construction implanted for vertebras fixations that could influence the electrical field and alter the physiological effects of the stimulation. There is a shortage of studies providing the evidence of influence the implanted materials on electrophysiological outcomes. Potential influence of the metal construction should be considered when using tSCS in SCI patients and further electrophysiological and computer simulation studies are required to investigate this in detail.

## Conclusion

The results of this work demonstrate that the afferent flow during positional tests and rehabilitation therapy can provide necessary excitation of spinal cord circuitries, helping in identification of neural connections, which can be further enhanced with rehabilitation and neuromodulation therapy. Considering that up to 80–90% of patients with clinically complete SCI have discomplete injury (Moss et al., 2011), it is expected that results of this study will provide significant background for a larger SCI population. The results of this case report emphasize the importance of evaluation with positional changes and reinforcement maneuvers during the assessment of SCI and could be important for future clinical trials and for assessments of patients with clinically complete SCI.

## Data Availability Statement

All datasets generated for this study are included in the article/Supplementary Material.

## Ethics Statement

The studies involving human participants were reviewed and approved by Kazan Federal University Institutional Review Board (Review board decision December 4th, 2017, protocol No. 7). The patients/participants provided their written informed consent to participate in this study. Written informed consent was obtained from the individual(s) for the publication of any potentially identifiable images or data included in this article.

## Author Contributions

AM and IL designed model framework. AM, EF, SS, and IL conducted the data collection and electrophysiology experiments. AM, EM, CC, JC, PG, TB, and IL analyzed the data and worked on the manuscript. IL approved the final version of the manuscript. All authors read and helped to improve the manuscript.

## Conflict of Interest

The authors declare that the research was conducted in the absence of any commercial or financial relationships that could be construed as a potential conflict of interest.

## Supplementary Material

VIDEO S1 Effect of Jendrassik maneuver on toes movements in supine and vertically suspended (100% body weight support).
