# Supplementary material for: Supraspinal and Afferent Signaling Facilitate Spinal Sensorimotor Network Excitability After Discomplete Spinal Cord Injury: A Case Report
Source: Front Neurosci. 2020 Jun 22;14:552. doi: 10.3389/fnins.2020.00552 (PMC7323764; doi:10.3389/fnins.2020.00552)
Supplement: Supplementary file 2 [file Data_Sheet_1.docx]

**Methods**

1. Electrophysiological assessment

The following electrophysiological assessment techniques were implemented in this study:

*I. Spinal cord somatosensory evoked potentials (SSEP):* Electrical pulses (Neuro MEP-8, Neurosoft, Ivanovo, Russia) were delivered to the tibial nerve bilaterally at the ankle area. Stimulation intensities corresponded to the visual threshold of the motor response of the muscles (flexion of the toes). Stimuli consisted of monophasic rectangular electrical pulses of 0.2 ms duration at 3 Hz and 1.5x of threshold of visual motor response. SSEPs were recorded at five locations (popliteal region, L2-3, Th11-12, Th8-9, and Cz-Fpz) (Fig. 2B) and an average response was calculated from 800 consecutive stimulus pulses (Fig. 2B). All test of SSEP was made before rehabilitation therapy.

*II.* *M/H-response:* Evaluation of M/H response was performed by stimulation of the right and left posterior tibial nerves in popliteal region, using a stainless-steel bipolar electrode. Responses were recorded from the soleus muscle with bipolar. The stimulation frequency was 0.1 Hz, the pulse duration 1 ms, and intensity from 2 to 30 mA. Stimulation intensity was gradually increased in steps of 1 mA until the amplitude of M-response in the soleus muscles no longer increased. The stimulation intensity required to evoke the maximum amplitude of the H-reflex was determined for each side.

*III. Spinally evoked motor potentials:* SEMP were recorded, using surface EMG electrodes (Kendall, Meditrace – 100; Ag/AgCl, diameter of 22 mm) from rectus femoris (RF), medial hamstring (MH), tibialis anterior (TA), and soleus (SOL) muscles on both sides. To evoke SEMPs, active gel adhesive electrodes (TensCare, CM25, diameter of 25 mm) were placed at the midline, in between the Th9-Th10, Th10-Th11, Th11-Th12, Th12-L1, and L1-L2 spinous processes, and two reference electrodes (4x2 cm) were placed over the lower abdominal area. Electrical stimulation (Neuro MEP-8, Neurosoft, Ivanovo, Russia) was performed with monophasic rectangular pulses with pulse duration of 1 ms every 10 seconds. Stimulation intensity was increased from 30 to 100 mA or to the maximum tolerable intensity. Ten stimuli were delivered at each stimulation intensity. At 100mA of stimulation amplitude, three paired pulses, each at an interstimulus interval of 50 ms, were applied. The effect of paired pulses stimulation was assessed to test the presence of post-activation depression and hence verify the reflex nature of the SEMP (Hofstoetter et al., 2018). If the tSCS activities the motor fibers the second response would be present.

*IV. Reinforcement and positional maneuvers*: The influence of supraspinal and afferent information on SEMP was evaluated with reinforcement maneuver (Jendrassik maneuver) and by body position changing. Jendrassik maneuver (JM) was performed in the following manner: the subjects clasped the fingers of both hands in a "lock" in front of the chest, and made prolonged attempt to break this "lock". At the beginning of the study, the participant was trained to perform JM. Beep sound was used as an audio signal to start JM. The first experimental session studied changes of the SOL H-reflex characteristics bilaterally under the action of JM. Subject is positioned supine on the couch with feet suspended. Recruitment curves were constructed by plotting the magnitude of the M-response and H-reflex against increasing stimulation intensity (see more in section II). Then, to assess the effects of the JM on the H-reflex amplitude, stimulation intensity was identified such that produced minimally M-response and H-reﬂexes with an amplitude ranging from 30 – 50 % of H-max. Second experimental session studied changes in the amplitude of the SEMPs evoked by tSCS under the action of JM at different body positions (supine vs upright position). **SEMPs were recorded with the tSCS at the Th12-L1 level in supine position and then the subject was placed in a body weight support system in upright position to repeat the test.** To optimize afferent feedback during testing and training we used dynamic BWS system to provide the necessary support for the subject to perform required tasks, that was less than 30% most of the time (Apte et al., 2018). To demonstrate the effect of transition from supine to **vertically suspended position** and toe movement in response to Jendrassik maneuver, subject was suspended in upright position with 100% body weight support (see Video 1, Supplementary materials). SEMPs were recorded on the bilaterally at the most intensity of the stimuli (100mA).

Physical and rehabilitation therapy

Following initial evaluations, the participant received standard rehabilitation program for mobility and self-care skills. Within this routine, the main component of the training was 65 sessions during 16 weeks (Fig. 1C) of physical therapy and motor rehabilitation. Subject with SCI did not receive any motor training apart from these sessions. Each session included stretching exercises up to 10 minutes to warm up, then functional training (passive lower extremities exercise and attempts to perform voluntary movements (flexion\extension) of each separate joint of legs) both in supine and sitting positions. In addition, 20 minutes of training on a treadmill with partial body weight support to train passive cycling. The trainers were assisted trunk and lower extremity kinematics and limb loading during stepping every session. Weight support and treadmill speed were adjusted to enable training at speeds 0.89 m/s – 1.34 m/s and at the maximum body weight allowed with minimal force need by the trainers to assist during stance. After 16 weeks of therapy, the subject with SCI repeated the same electrophysiological evaluation.

1. Electrophysiological recording and data processing

Surface EMG recordings (Neuro MEP-8, Neurosoft, Ivanovo, Russia) were performed bilaterally from RF, MH, TA, and SOL muscles, using a bipolar electrode (diameter 24 mm) with a fixed inter-electrode distance of 20 mm. Data were sampled at 5 kHz and post-processed using OriginPro 8.1 (OriginLab Corporation, Northampton, USA) and SigmaStat 4.0 (Systat Software, San Jose, CA, USA). SEMP were recorded from all tested muscles, averaged from 10 stims, and analyzed in the window of 20 ms to 100 ms from stimulus pulse onset. During spinal cord stimulation, SEMPs were induced in the leg muscles and their peak‐to‐peak amplitude was calculated within 0 to 30 ms time window for the monosynaptic component of the SEMP and 30 to 100ms for polysynaptic responses. The threshold, amplitude, and latency of each response were identified for analysis. The threshold of SEMPs was defined as the minimum voltage of the stimulus necessary to trigger minimum amplitudes of evoked potentials.

1. Statistical analyses

All results are reported as means ± standard error of the mean. Statistically significant differences were determined using one-way repeated-measure ANOVA (Student-Newman-Keuls), Mann Whitney U Test, and Student t-test. The statistical significance was set at p˂0,05.
